# Simultaneous detection of diethylstilbestrol and estradiol residues with a single immunochromatographic assay strip

**DOI:** 10.1002/fsn3.2127

**Published:** 2021-01-20

**Authors:** Zehua Xu, Tieqiang Sun, Hongwei He, Wentao Liu, Longxing Fan, Lingdi Zhao, Xinglin Wu, Zhenyu Han, Yingcun Zhang, Qiangqiang Wang, Baoan Ning, Zhixian Gao

**Affiliations:** ^1^ Tianjin Institute of Environmental and Operational Medicine Tianjin China

**Keywords:** diethylstilbestrol, estradiol, gold nanoparticles, immunochromatographic assay

## Abstract

An immunochromatographic assay (ICA) based on competitive format was developed and validated for simultaneously rapid and sensitive detection of diethylstilbestrol (DES) and estradiol (E_2_) in milk and tissue samples. For this purpose, two monoclonal antibodies raised against those two estrogens were conjugated to gold nanoparticles and were applied to the conjugate pads of the test strip. The competitors of the DES‐BSA/E_2_‐BSA conjugates were immobilized onto a nitrocellulose membrane at two detection zones to form T_1_ and T_2_, respectively. The immunochromatographic assay had a visual detection limit of DES at 30 ng/g in milk powder, 25 ng/g in liquid milk, and 25 ng/g in shrimp tissue, respectively, and the results can be judged within 7–10 min. The visual detection limit of E_2_ was 75 ng/g in milk powder, 65 ng/g in liquid milk, and 60 ng/g in shrimp tissue, respectively, and the results can be judged within 3–4 min. It had advantages in easy operation without requiring sophisticated equipment and specialized skills. By testing thirty milk and shrimp tissue samples from the local market, the method was compared with the HPLC‐MS / MS method, and there was no statistical difference between the two methods. Furthermore, the immunochromatographic assay had good specificity, simple procedure, and low cost. This protocol was well suited for the food safety monitoring and early warning.

## Graphical abstract

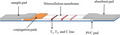

## 1 INTRODUCTION

Diethylstilbestrol (DES) and estradiol (E_2_) belong to estrogens and are sold as a kind of estrogen modulators. But they are found in water and soil due to their unreasonable applications in industry and agriculture. They are also found in animal food products on account of being used to fatten animals (Huang et al., 2015; Yang et al., 2015). Furthermore, the long half‐life of them can cause accumulation in animal products, such as liver, muscle, and kidney. Then, they can enter human body through the food chain (Gadd et al., 2010) and affect the hormone level, even increasing the incidence of cancer and tumors (Adami et al., 2012; Eliassen et al., 2012; Jacobsen et al., 2005). Subsequently, usage of estrogens in food is strictly banned in most countries and areas around the world, such as China, the United States, and Europe. However, they are often illegally used as a growth promoter in cows, calves, sheep, and fish. Thus, an efficient method for the determination of DES and E_2_ needs to be established to monitor their residues in animal product food in order to ensure the safety of the food supply.

To monitor these residues, several analytical methods including physicochemical and immunoassays have been developed over the past decade, but they still have significant drawbacks. Physicochemical procedures such as high‐performance liquid chromatography (HPLC) (Gañán et al., 2013; Kumar et al., 2014; Patrolecco et al., 2013), liquid chromatography/mass spectrometry/mass spectrometry (LC‐MS/MS; Nguyen et al., 2011; Regal et al., 2013; Szarka et al., 2013), and gas chromatography/mass spectrometry (GC‐MS; Franke et al., 2011) can detect estrogens at low concentration (Yu et al., 2013), but drawbacks including high costs and complicated sample pretreatment arise simultaneously. Immunoassays, relying on the specificity of antibody molecules to their corresponding antigens, have widely been applied in biochemical studies (Azzazy et al., 2006; Honda et al., 2005). Various methods have been developed for measuring estrogens in serum based on immunoassay methods, such as enzyme‐linked immunosorbent assays (ELISA) (Zhao et al., 2004), fluorescence immunoassays (Yan et al., 2005), radio‐immunoassays (Chester et al., 1991), and chemiluminescence immunoassays (Lin et al., 2004). The main disadvantage of these strategies is the use of secondary and tertiary antibodies in which the enzyme can be ligated. Moreover, these determinations are a multistep process, require complex and expensive instruments, and usually take a long time (Gao et al., 2018).

Hence, detection methods of estrogens with short analysis time, simple procedure, and low cost are urgently needed for the food safety monitoring and early warning. Current research in the laboratory is focused on the development of rapid point‐of‐care (POC) tests such as immunochromatographic assay (ICA; Buchinger et al., 2013; Zhang et al., 2013). This technology displays remarkable advantages: fast, simple, and low cost. Researches about ICA methods have been reported to detect various chemical contaminants in food. However, most of these studies are limited to the detection of only a single target (Jiang et al., 2011).

**FIGURE 1:**
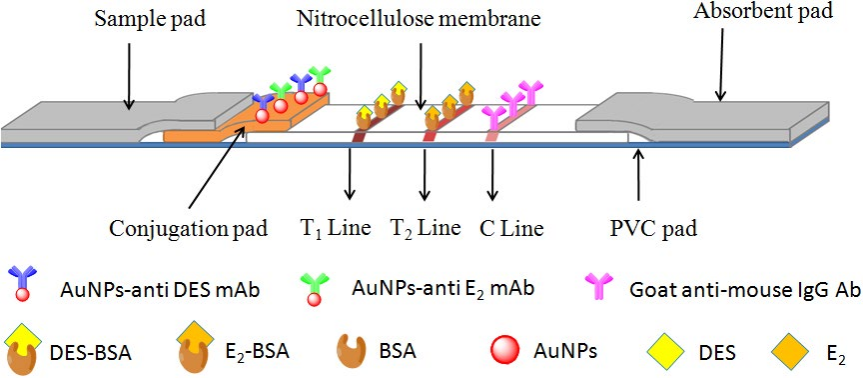
Schemes of the immunochromatographic assay strip

Now, we have developed a new ICA which can simultaneously detect diethylstilbestrol and estradiol residues on a single immunochromatographic assay strip. The scheme of test strips is shown in Figure 1. The device consists of a nitrocellulose membrane on which a goat anti‐mouse IgG (C line), and DES‐BSA and E2‐BSA (T1 and T2 capture line) are immobilized. Fixed at one end of the membrane is a binding pad containing a colloidal gold antibody conjugate. A sample pad overlays the conjugation pad. The other end of the membrane is an absorbent pad which performs to facilitate capillary flow through the ICA strips. The entire device is fixed to a PVC pad. Sample is added at the sample port, absorbed into the sample pad, and drawn by capillary action force through the conjugation pad. In the absence of analyte, the colloidal gold–antibody conjugates flow through the nitrocellulose membrane, resulting in a red line obvious to visual inspection on T1, T2, and C band. If the sample contains sufficient targets, T1 and T2 band would not appear in the corresponding location. But in every assay, the C line (control line) should always be observed to ensure that the system is working effectively.

## 2 MATERIALS AND METHODS

### 2.1 Chemicals and reagents

Chloroauric acid (HAuCl4.3H2O), sodium citrate (C6H5Na3O7.2H2O), bovine serum albumin (BSA), DES, and E2 were purchased from Sigma‐Aldrich (USA). The goat anti‐mouse antibody was obtained from Zhongshan Company (China). Diethylstilbestrol–BSA (DES‐BSA), estradiol–BSA (E2‐BSA), and the monoclonal antibody against DES (mAb F6B3‐4) and E2 (mAb F12C3‐2) were prepared in our laboratory. Nitrocellulose membranes (HAHY 00010), glass fiber conjugate pad (GFCP001050), and absorbent paper (CFSP001750) were purchased from Millipore Corporation. Other reagents were of analytical grade or higher.

### 2.2 Apparatus

BioJet Platform HM3010 (Shanghai Kinbio Technology Company), HPLC‐MS/MS (Agilent 6,410), Camera (Sony α700), and UV‐VIS Spectrophotometers (Purkinje General Instrument Co., TU‐1901) were used.

### 2.3 Synthesis of colloidal gold

**FIGURE 2:**
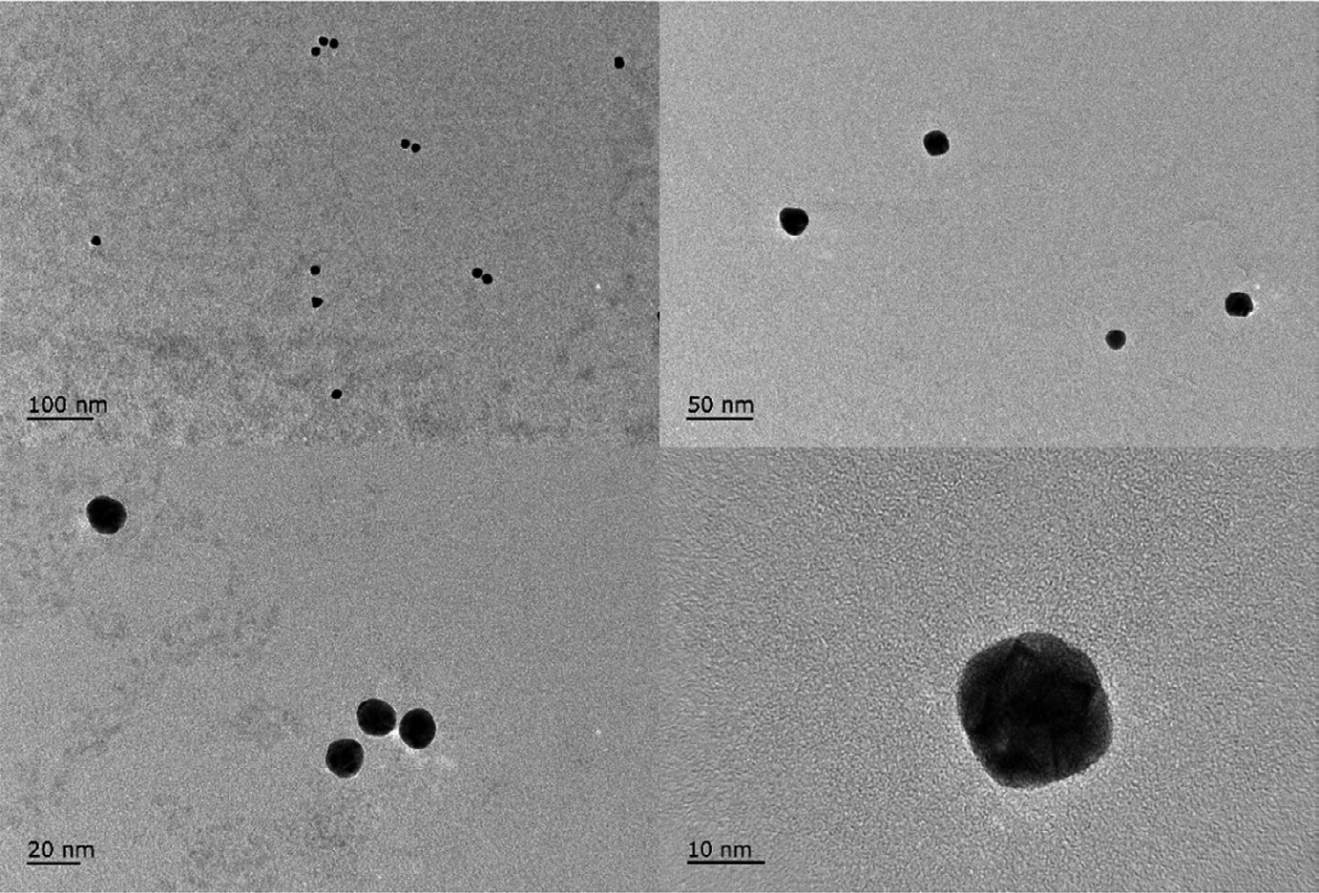
TEM characterization of colloidal gold

Colloidal gold was prepared using a previously reported method with some modifications (Song et al., 2016). Briefly, 100 ml of 0.01% (w/w) aqueous solution of chloroauric acid (HAuCl4 0.3H2O) in a beaker (500 ml volume) was boiled under stirring, and 1.8 ml of 1% trisodium citrate was then quickly added to the solution. The color of the solution turned into deep blue and gradually changed into red. After boiled within 7–10 min, the colloidal gold solution was cooled till room temperature. Water was added to form a final volume of 100 ml, and 0.02–0.05 g PEG 2000 was added in the end. The colloidal gold solution was stored at 4°C and should not be frozen (Figure 2).

### 2.4 Synthesis of Colloidal Gold–Monoclonal Antibody Conjugate Probe (CG‐mAb)

**FIGURE 3:**
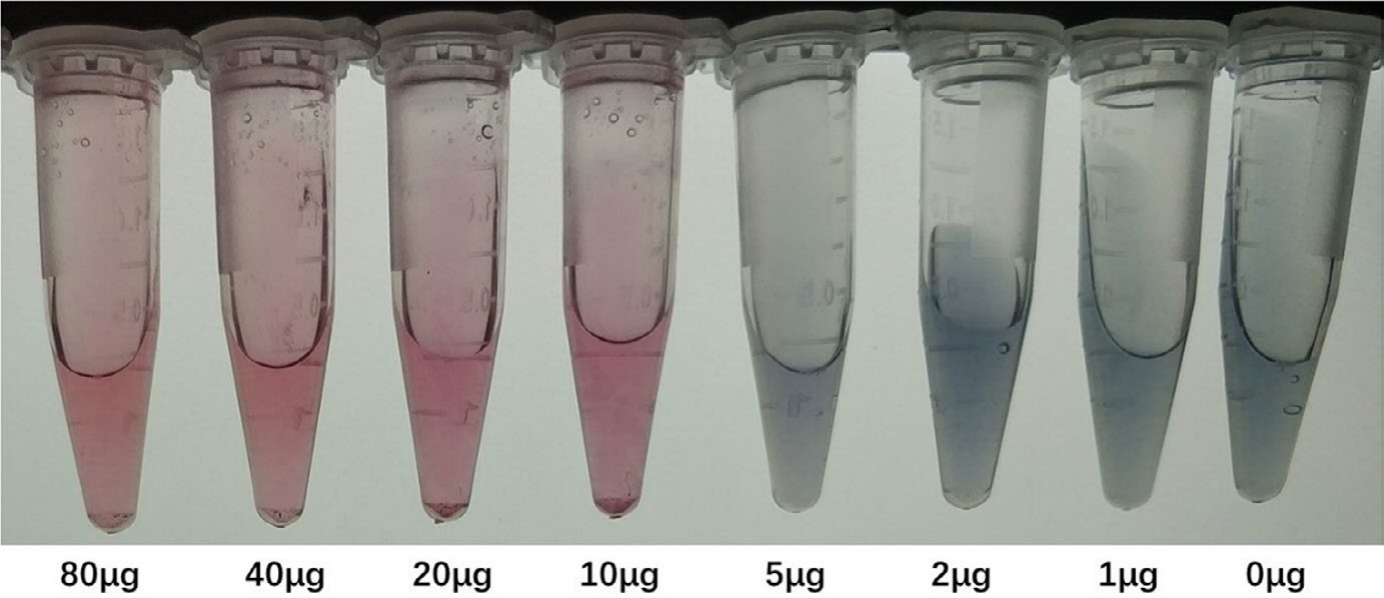
Mey's stability assay determined the amount of purified monoclonal antibody (F6B3). F12C3‐2 uses the same method to determine the amount of addition

Respectively absorb 1 ml of the prepared colloidal gold solution into 8 Eppendorf centrifuge tubes. The pH was adjusted to 7.0 with 0.1 mol/L K2CO3, and 0, 1, 2, 5, 10, 20, 40, and 80 μg of purified mAb (F6B3) were added, and after standing for 5 min, 0.1 ml of 10% NaCl was added. When the color of the reaction system changes from red to blue, the antibody concentration in the system is the smallest antibody concentration. The ultraviolet‐visible spectrum is scanned in the range of 400 ~ 600 nm to get the maximum absorption wavelength. The UV‐visible spectrophotometric solution was used to scan in the range of 400–600 nm to obtain the maximum absorption wavelength. At this wavelength, the absorbance of the complex formed by different antibody concentrations was measured, and the absorbance value was plotted against the antibody concentration. Continue to add antibody on the basis of the minimum antibody concentration, and the absorbance hardly changes. On this basis, an increase of 20% is the optimal monoclonal antibody (F6B3) amount (Figure 3).

Two monoclonal antibodies named F6B3‐4 and F12C3‐2 were raised against DES and E2, respectively. These antibodies prepared in our laboratory were purified from mouse ascitic fluid using caprylic acid and ammonium sulfate method. Each antibody was conjugated to gold nanoparticles as follows: 30 μg of the purified mAb (F6B3‐4 or F12C3‐2) was added into 2 ml gold colloid solution previously adjusted to pH 7.0 with 0.1 mol/L K2CO3. The mixture was stirred for 40 min, and then, 40μl 5% BSA was added to block unreacted gold colloid. After standing for 30 min, the solution was centrifuged at 400 *g* for 15 min and the precipitation was removed. Then, the resulting supernatant was centrifuged at 8,000 *g* for 30 min. The resulting precipitation was suspended in 20 ml 0.1 mol/L PBS (pH 7.4) containing 1% BSA and 0.05%–0.1% NaN3. The conjugates of colloidal gold–DES–mAb and colloidal gold–E2–mAb were stored at 4°C until use.

### 2.5 Preparation of the test strips

The strip consisted of four sections including sample pad, conjugation‐released pad, nitrocellulose membrane, and absorbent pad. Briefly, the sample pads (glass fiber conjugate pad) were treated with blocking buffer pH 7.4 (0.01 mol/L phosphate buffer containing 2.97 g NaH2PO4, 28.99 g Na2HPO4, 9.00 g NaCl, 1% BSA, and 0.25% Tween‐20, 1 L H2O) and then dried at 37℃ overnight.

The conjugation‐released pad was prepared by a jet of the mixture of colloidal gold–antibodies conjugated (10/10, v/v) at a rate of 2.0 μl/cm using a BioJet Platform, and dried at 37℃ for 24 hr.

Nitrocellulose membrane was treated with DES‐BSA, E2‐BSA, and goat anti‐mouse antibody. They were 1.0 mg/ml and sprayed into the nitrocellulose membrane at a rate of 1.0 μl/cm to form T1, T2, and C lines, respectively. After spotted, the membranes were dried at 37°C for 24 hr. Absorbent pads were untreated.

Subsequently, the sample pad, detector pad, nitrocellulose membrane, and absorbent pad were cut into 4‐mm test strip and laminated into a sheet of plastic backing orderly. The test strip was then stored at 4–30°C in plastic bags.

### 2.6 Detection of DES and E_2_ By a single immunochromatographic assay strip

Experiments were carried out to determine the detection limit of the test strips for DES and E2. Standard solution of DES in 0.01 mol/L phosphate buffer at concentrations 0, 10, 50, 100, 200, 300, and 400 ng/ml was analyzed with the test strip. The procedure of E2 is as the same, with standard solution in 0.01 mol/L phosphate buffer at concentrations 0, 200, 300, 400, 500, 600, and 700 ng/ml. Then, DES and E2 were mixed together to prepare a serial standard solution with final concentrations as shown in Table 1.

**TABLE 1:** Accuracy of the strip (*n* = 10)

	Concentration(ng/ml)	Number	
		Positive	Negative
DES	0	0	10
	50	0	10
	100	0	10
	200	10	0
	300	10	0
E_2_	0	0	10
	300	0	10
	400	0	10
	500	9	1
	600	10	0

### 2.7 Precision and reproducibility study

Standard solution of DES in 0.01 mol/L phosphate buffer at 0, 50, 100, 200, and 300 ng/ml was analyzed ten times (*n* = 10) for the intraday assay and daily for ten successive days for the interday assays. The procedure of E2 is as the same procedure as DES with standard solution in 0.01 mol/L phosphate buffer at 0, 300, 400, 500, and 600 ng/ml.

### 2.8 Sample pretreatments for test strip analysis

Two grams (2 ± 0.05 g) of milk samples was mixed with 6 ml of ethyl acetate in 10‐mL centrifuge tube and then left to stand for 2–3 min (Note: no vigorous vibration or vortex oscillation to prevent sample from emulsification!). This was followed by centrifugation at 3,000 *g* for 10 min. Afterward, 2.5 ml of supernatant was dried under a gentle flow of nitrogen. The residue obtained was then resuspended in 100 μl of 0.01 mol/L phosphate buffer (pH 7.4) by vortex oscillation before detected by the test strips.

Two grams (2 ± 0.05 g) of tissue samples was mixed with 6 ml of acetonitrile: acetone = 3:2 (v/v). After drastic vortex oscillation at 2000 rpm for 20 min, the solution was centrifuged at 3,000 *g* for 5 min. The 2.5 ml supernatant was dried under a gentle flow of nitrogen. The residue obtained was then resuspended in 200 μl of 0.01 mol/L phosphate buffer (pH 7.4) by vortex oscillation before detected by the test strips.

### 2.9 Detection of local market samples by ICA and HPLC‐MS/MS

Thirty milk samples and seven shrimp tissue samples were randomly collected from local markets in Tianjin (China). These samples were analyzed by the strip and HPLC‐MS/MS (Frens, 1973; Lin et al., 2007). The chromatographic separation was performed on an Agilent C18 column (2.1 × 50 mm, 2.7 µm). Mobile phase A consisted of 0.1% acetic acid in water, and mobile phase B was acetonitrile. The flow rate was 0.25 ml/min with 75% B. The injection volume was set at 10 µl. The mass spectrometer detector was operated in negative‐ion mode. The retention time of DES was 1.9 min and that of E2 was 1.3 min. The data were acquired in the negative selective reaction monitoring (MRM) mode, using the following conditions: m/z 267.2 → 251.1 fragmentor 137 V collision energy 25 V and m/z 267.2 → 236.9* fragmentor 137 V collision energy 25 V for DES; and m/z 271.1 → 183* fragmentor 170 V collision energy 42 V and 271.1 → 143.2 fragmentor 170 V collision energy 42 V for E2.

## 3 RESULTS AND DISCUSSION

### 3.1 Detection of DES and E2 By the test strip

A serial standard solution of DES, ranging from 0 to 400 ng/ml, was prepared in a phosphate buffer, and the assay was carried out by the addition of the standard solution of DES (100 μl) to the sample pad. A visible red line (T1 line) was resolved in 7–10 min. As shown in Figure 2a, T1 line did not appear when the concentration of DES was greater than or equal to 200 ng/ml.

A serial standard solution of E2, ranging from 0 to 700 ng/ml, was prepared in a phosphate buffer, and the assay was performed by the application of standard solution of E2 (100 μl) to the sample pad. A visible red line (T2 line) was resolved in 3 ~ 4 min. As shown in Figure 2b, T2 line would appear if concentration of E2 was less than 500 ng/ml.

**FIGURE 4:**
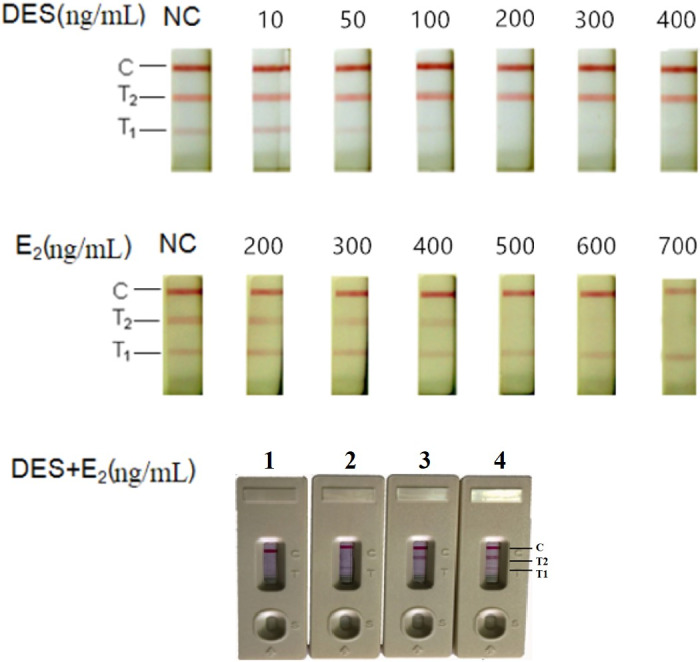
Detection of DES and E2 By the ICA strip. (a) A serial standard solution of DES from 0 to 400 ng/ml, (b) a serial standard solution of E2 from 0 to 700 ng/ml, and (c) the mixed solution of DES and E2 at final concentration 300 ng/ml DES and 600 ng/ml E2 (sample 1), 50 ng/ml DES and 600 ng/ml E2 (sample 2), 300 ng/ml DES and 200 ng/ml E2 (sample 3), and 50 ng/ml DES and 200 ng/ml E2 (sample 4)

As shown in Figure 4a, when DES, ranging from 0 to 400 ng/ml, flowed through the test strip, the T2 line was still visible which indicated that DES did not react with anti‐E2 antibody. On the other hand, as shown in Figure 4b, the T1 line was still observed even when standard solution of E2 added into sample pad reached 700 ng/ml. So, the monoclonal antibody for DES and E2 demonstrated high specificity, showing no reactivity between DES and E2. They could be used together in a single immunochromatographic assay strip capable of detecting DES and E2 simultaneously. These results were demonstrated in Figure 4c. The results of sample 1 showed that T1, T2, and C lines turned red because the concentration of DES (50 ng/ml) was less than the detection limit of DES (200 ng/ml) and the concentration of E2 (200 ng/ml) was less than the detection limit of E2 (500 ng/ml). When the concentration of DES was more than 200 ng/ml or the concentration of E2 was above 500 ng/ml, corresponding test line would not appear such as the results of sample 2 or sample 3. If the concentration of DES and E2 was more than the detection limit of them, T1 and T2 lines would not be observed.

### 3.2 Accuracy

The reproducibility and stability of the strip were tested using standard solution of DES at concentrations of 0–300 ng/ml and standard solution of E2 at concentrations of 0–600 ng/ml (Table 1). The detection method of DES has 100% (50/50) reproducibility, and the detection method of E2 has 98% (49/50) reproducibility. The results showed that the ICA has high accuracy and good repetition.

### 3.3 Specificity

Table 2 showed the results of specificity experiments of the test strip. It suggested that when the analyte concentration was above the data listed in Table 3, the corresponding test line would disappear which resulted from the reaction of the analyte with the antibody. Take dienestrol for example, the test line for DES could not be observed when the concentration of dienestrol was above 50 µg/ml, and the test line for E_2_ would disappear when above 5 µg/ml. However, the concentration of E_2_/DES in real samples scarcely reached 5/50 µg/ml. Hence, dienestrol would not interfere with the detection of E_2_/DES, which indicated that the ICA strip had well specificity.

**TABLE 2:** Results of specificity experiments of the test strip

	Estrogens(µg/ml)					
	DES	E_2_	Estrone	Dienestrol	Progesterone	Hexestrol
T1 lines disappear		>50	>50	>50	>50	>1
T2 lines disappear	>50		>50	>5	>50	>5

**TABLE 3:** Limit of detection of DES and E_2_ in spiked food using the ICA

	DES (ng/g)	E_2_ (ng/g)
Milk powder	30	70
Liquid milk	25	65
Shrimp tissue	25	60

### 3.4 Detection of DES and E_2_ in milk powder, liquid milk, and shrimp tissue

To study the performance of the DES and E2 ICA strip in real samples, we spiked milk powder, liquid milk, and shrimp tissue with DES and E2 to form the final concentration at 20, 30, 60, 80, and 100 ng/g. As shown in Table 3, the limit of detection of DES was 30, 25, and 25 ng/g in milk powder, liquid milk, and shrimp tissue, respectively. The limit of detection of E2 was 70, 65, and 60 ng/g accordingly. The limit of detection in milk powder was higher because it may contain more fat.

### 3.5 Comparison between HPLC‐MS/MS and ICA for the analysis of samples from local markets

Table 4 summarized the detection results obtained by HPLC‐MS/MS and ICA. Three samples were found DES positive, and five samples were E2 positive by the ICA methods. With HPLC‐MS/MS method, two samples were DES positive and six samples E2 positive. The data would be analyzed with correspondingly statistical methods. The ICA method of DES had a sensitivity of 100% (2/2), a specificity of 97.1% (34/35), a positive predictive value of 66.7% (2/3), a negative predictive value of 100% (34/34), and *κ* = 0.794 > 0.75. The ICA method of E2 had a sensitivity of 66.7% (4/6), a specificity of 96.8% (30/31), a positive predictive value of 80% (4/5), a negative predictive value of 93.8% (30/32), and *κ* = 0.680 > 0.4. According to statistic theory, there was good congruence in the two methods (ICA and HPLC‐MS/MS), and the ICA method of DES had better correlation than that of E2 (kappa 0.794 > 0.680). Because the method of DES was of higher sensitivity.

**TABLE 4:** Comparison between ICA and HPLC‐MS/MS for the analysis of DES and E2 from local markets

Number	Results by HPLC‐MS/MS		
	Positive	Negative	Total
Results by ICA			
DES			
Positive	2	1	3
Negative	0	34	34
Total	2	35	37
E2			
Positive	4	1	5
Negative	2	30	32
Total	6	31	37

## 4 CONCLUSION

Here, we reported the design and use of a single ICA strip capable of detecting and distinguishing between DES and E2. The ICA demonstrated the greater sensitivity for DES, detecting as little as 30 ng/g in milk powder, 25 ng/g in liquid milk, and 25 ng/g in shrimp tissue, respectively. E2 could be detected down to 75 ng/g in milk powder, 65 ng/g in liquid milk, and 60 ng/g in shrimp tissue, respectively. The development of new ICA made available powerful and high‐throughput detection and screening methods, which was more efficient than the traditional one. Further work would focus on the integration of more targets on one ICA strip and quantitative detection. Thus, this strip would play an important role in monitoring multiple residues in food.

## CONFLICTS OF INTEREST

No potential conflicts of interest relevant to this article were reported.
